# Treatment of Spinal Tuberculosis by Debridement, Interbody Fusion and Internal Fixation via Posterior Approach Only

**DOI:** 10.1111/os.12228

**Published:** 2016-03-30

**Authors:** Ming‐xing Tang, Hong‐qi Zhang, Yu‐xiang Wang, Chao‐feng Guo, Jin‐yang Liu

**Affiliations:** ^1^ Department of Spine Surgery Xianya Hospital, Central South University Changsha China

**Keywords:** Spinal tuberculosis, Posterior approach, Interbody fusion, Internal fixation

## Abstract

Surgical treatment for spinal tuberculosis includes focal tuberculosis debridement, segmental stability reconstruction, neural decompression and kyphotic deformity correction. For the lesions mainly involved anterior and middle column of the spine, anterior operation of debridement and fusion with internal fixation has been becoming the most frequently used surgical technique for the spinal tuberculosis. However, high risk of structural damage might relate with anterior surgery, such as damage in lungs, heart, kidney, ureter and bowel, and the deformity correction is also limited. Due to the organs are in the front of spine, there are less complications in posterior approach. Spinal pedicle screw passes through the spinal three‐column structure, which provides more powerful orthopedic forces compared with the vertebral body screw, and the kyphotic deformity correction effect is better in posterior approach. In this paper, we report a 68‐year‐old male patient with thoracic tuberculosis who underwent surgical treatment by debridement, interbody fusion and internal fixation via posterior approach only. The patient was placed in prone position under general anesthesia. Posterior midline incision was performed, and the posterior spinal construction was exposed. Then place pedicle screw, and fix one side rod temporarily. Make the side of more bone destruction and larger abscess as lesion debridement side. Resect the unilateral facet joint, and retain contralateral structure integrity. Protect the spinal cord, nerve root. Clear sequestrum, necrotic tissue, abscess of paravertebral and intervertebral space. Specially designed titanium mesh cages or bone blocks were implanted into interbody. Fix both side rods and compress both sides to make the mesh cages and bone blocks tight. Reconstruct posterior column structure with allogeneic bone and autologous bone. Using this technique, the procedures of debridement, spinal cord decompression, deformity correction, bone grafting, and internal fixation can be completed with only one incision and surgical position, and the deformity correction efficiency is higher than anterior surgery.

## Introduction

Bone and joint tuberculosis (TB) accounts for 13% of TB cases; 50% of these are in the spine.^1^ The incidence of TB has increased globally in recent years because of large‐scale migration, increase in number of patients with HIV infection, emergence of antibiotic‐resistant strains and other reasons. Patients with spinal TB often have severe nerve damage, spinal instability and kyphotic deformity and therefore usually require surgical treatment. The aims of such treatment are to eradicate the TB lesion, relieve spinal nerve compression, reconstruct spinal stability and correct spinal deformity. For spinal destruction by TB foci in the anterior and middle columns, anterior debridement, bone grafting and either anterior or posterior internal fixation have been performed by many researchers.^2^, ^3^, ^4^, ^5^, ^6^, ^7^, ^8^ However, because of the complex anterior structure, this approach is associated with many complications.^9^, ^10^, ^11^ Furthermore, combined posterior and anterior procedures lead to greater blood loss, increased operating time, prolonged anesthesia, more hospitalization expenses, and increased mortality and complications. In addition, exposure of the upper thoracic and lumbosacral segments is a major challenge for surgeons.

Because tuberculous lesions always involve the anterior and middle columns of the spinal vertebrae, many researchers have concerns about whether posterior surgery can accomplish focal debridement completely with its limited visual field, how it may affect the stability of the spine and whether it may cause intraspinal infection. We have designed a new posterior approach for treating spinal TB that effectively addresses the above problems. From January 2006 to January 2012, 290 patients with spinal TB have undergone surgery comprising debridement, interbody fusion and internal fixation *via* a posterior approach only in our hospital. In this paper, we present a typical case of a 68‐year‐old male patient who was admitted to our hospital with thoracic TB and underwent surgical treatment comprising debridement, interbody fusion and internal fixation *via* a posterior approach only to illustrate the clinical efficacy and feasibility of this new surgical procedure.

## Case Presentation

### Patient

A 68‐year‐old male patient was admitted to our hospital with chest pain for 6 months and lower limb fatigue for 1 month. There had been no obvious cause for the onset of back pain 6 months ago. This pain was particularly evident at night and had become more severe and accompanied by lower limb fatigue 1 month previously. He had received no anti‐TB therapy before admission. X‐rays, MRI and CT of spine, routine blood tests, erythrocyte sedimentation rate (ESR), C‐reactive protein (CRP) concentrations and hepatorenal function were examined. He had a white blood cell count was 5.7 × 10^9^/L, ESR 87 mm/1h, CRP concentration 21.4 mg/L and normal hepatorenal function.

The patient consented to publication of data concerning his case.

### Physical Examination

Physical examination showed hypesthesia below the rib arch, Grade 1 lower limb muscle strength and Frankel Grade C neurological function. It also showed increased muscular tone in the lower limbs, knee and ankle hyperreflexia and positive sign for ankle clonus.

### Imaging Study

X‐ray films showed collapse of the T_8_ vertebrae and T_7–8_ intervertebral space. CT scan showed destruction of T_7_, T_8_ and the intervertebral space and sequestrum formation, especially on the left side. A paravertebral abscess was limited to T_7_ and T_8_ without an obvious gravitation abscess. The height of the T_8_ vertebral body was markedly reduced and the dural sac and spinal cord severely compressed by an abscess posterior to the vertebral body.

## Treatment

The patient was administered the anti‐TB drugs isoniazid, rifampicin, ethambutol and pyrazinamide prior surgery. He underwent surgical debridement, interbody fusion and internal fixation *via* a posterior approach 7 days after starting anti‐TB therapy because his paralysis progressively worsened after commencing chemotherapy. He continued to receive anti‐TB chemotherapy for 18 months postoperatively.

### Surgical Technique

The patient was placed in a prone position under general anesthesia with somatosensory‐evoked potential monitoring. Extraperiosteal dissection at both non‐fusion and fusion levels was performed through a midline incision. The posterior spinal construction was exposed, including the spinous processes, lamina, facet joints and transverse processes and the range of exposure at the level of decompression expanded to include the costotransverse articulations and 3–5 cm of the medial ribs bilaterally. Pedicle screws or hooks were placed at one or two levels superior and inferior to the level of decompression under C‐arm fluoroscopy guidance.

**Figure 1:**
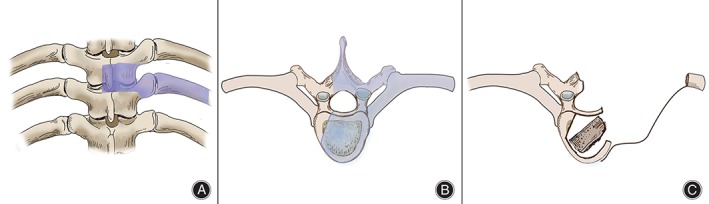
Diagrammatic representation of surgical management of spinal tuberculosis via a posterior approach only. (A) The range of excision viewed from behind, including the spinous process, facet joint on one side and lower costotransverse joint (with a small fragment of ribs if in thoracic segment). (B) The range of excision viewed axially. (C) Specially constructed titanium mesh cages or bone blocks are implanted in the interbody via the posterior approach after debridement.

**Figure 2:**
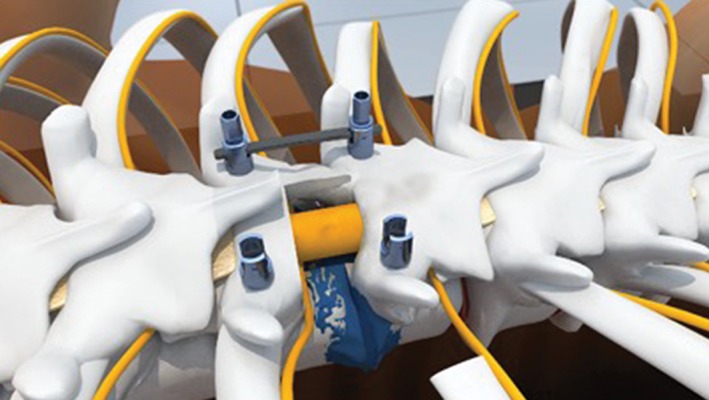
Video screen shot showed temporary fixation with one side rod and resection of the spinous process, facet joint on one side and the lower costotransverse joint with a small fragment of rib.

One side rod was fixed temporarily to avoid spinal cord injury during decompression and focal debridement. Lesion debridement to drain the prevertebral abscess and expose the diseased vertebral bodies was performed from the side with more bone destruction and a larger abscess (left). The facet joint was resected unilaterally and the lower costotransverse joints excised with a small fragment of adjoining rib if in the thoracic segment, retaining contralateral structural integrity^12^ (Figs 1, 2), while protecting the spinal cord and nerve root. Spatulas of various sizes and angles were used from a posterior approach to remove all lesions, including sequestra, abscesses and granulation tissues. The abscesses were drained by suction and curettage as thoroughly as possible.

**Figure 3:**
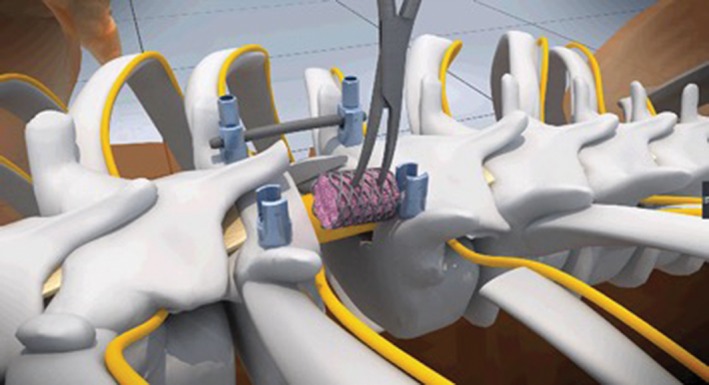
Video screen shot showing implantation of specially constructed titanium mesh cages into the interbody via a posterior approach only.

After distracting the intervertebral space, specially constructed titanium mesh cages or bone blocks were implanted into the interbody (Fig. 3). Both ends of the titanium mesh cages were filled with autologous bone with allograft or autogenous bone in the middle.^13^ Both side rods were then fixed and both sides compressed to tighten the mesh cages and bone blocks. After completion of internal fixation, debridement and interbody thoracic fusion were performed, and strip‐sized autogenous or allograft bone imbedded in the posterior body to fuse the segments that had been subjected to decompression and focal debridement. Streptomycin (1.0 g) and isoniazid (0.3 g) were deposited locally. The debrided material underwent bacterial culture and histopathologic examination.

### Postoperative Management

Antibiotics were given i.v. injection during the first postoperative week. The drainage tube was removed when the drainage volume was less than 20 mL/24 h. The patient was asked to wear a brace as soon as possible and then for at least for 6 months.^14^ The patient received anti‐TB chemotherapy with the four drugs mentioned above for at least 9 months and isoniazid, rifampicin and ethambutol treatment for another 3–6 months thereafter.

### Follow‐up

The patient was examined clinically and radiologically 3, 6 and 12 months after surgery and then at yearly intervals. X‐rays and blood tests were examined. Bone graft fusion was assessed using Bridwell's radiologic criteria.^15^ The patient had significant improvement in back pain after surgery and had achieved bony spinal fusion 6 months after surgery. His ESR and CRP had recovered to normal values by 3 months postoperatively.

## Discussion

### Spinal Tuberculosis

Spinal TB accounts for approximately half of all cases of musculoskeletal TB, which is more common in children and young adults. The incidence of spinal TB is increasing in developing nations, especially in China. Chemotherapy is a very effective way of controlling and treating TB and most individuals with spinal TB can be cured by conservative treatment. However, patients whose disease is not sensitive to anti‐TB chemotherapy and who develop progressive kyphosis, bone destruction or neurological impairment usually require surgical treatment.

The purpose of surgical treatment is debridement of focal TB, reconstruction of segmental stability, neural decompression and correction of kyphotic deformity. For the lesions mainly involved anterior and middle column of the spine, Hodgson *et al.* first reported their “Hong Kong operation” for treating spinal TB in 1960.^16^ With the development of instrumentation techniques, a one stage anterior procedure comprising debridement and fusion with internal fixation has become the most frequently performed surgical treatment for spinal TB.^5^, ^10^, ^17^, ^18^, ^19^ An anterior approach allows direct debridement, which facilitates focal debridement and nerve decompression, without destroying the spinal posterior column structure.^20^, ^21^ However, the anatomical structures encountered with an anterior approach are more complex, including major blood and lymphatic vessels, nerves and other important organs such as the lungs, heart, kidney, ureter and bowel. There is therefore a high risk of structural damage associated with such surgery.

### Posterior Approach Surgery

Because tuberculous lesions always involve the anterior and middle columns, many researchers have the following concerns about posterior surgery: (i) whether the surgery can accomplish focal debridement completely with its limited visual field; (ii) whether it will affect the stability of the spine; and (iii) whether it will cause intra‐spinal infection and central nervous system complications. We create adequate operating space by resecting the spinous process and facet joint on the more severely involved side and excising the adjacent costotransverse joint with a small fragment of ribs; this allows posterior decompression, anterior debridement and strut bone grafting without risking spinal cord injury because the spinal dura mater can be directly visualized. Relatively complete focal debridement, which requires removal of sequestra, granulation tissue and abscesses, can only be achieved *via* a posterior approach. We resect the facet joint unilaterally: the pedicle screw supplies enough support to maintain segmental stability of the spine in the early stages and solid stability is achieved when the bone fuses at a later stage. Because with a posterior approach the spinal canal is exposed to TB, some researchers are concerned about the possibility of intraspinal infection and central nervous system complications of TB infection, such as TB meningitis when removing TB foci *via* this approach.^22^, ^23^ In our series, no patients have developed TB meningitis, this possibly being attributable to initiating anti‐TB treatment preoperatively and continuing it postoperatively, inserting anti‐TB drugs locally intraoperatively and the biological membrane barrier provided by the spinal dura mater.

The posterior approach has some unique advantages over other surgical procedures. Firstly, the operative procedures of debridement, spinal cord decompression, deformity correction, bone grafting and internal fixation can all be completed *via* only one incision and in one position and better correction of deformity is achieved than with anterior surgery. Secondly, without one‐lung ventilation and with less operative trauma, there is less interference with the lungs, aorta and intestinal and therefore fewer postoperative complications such as atelectasis, lung infection, chylothorax, massive hemorrhage and paralytic ileus. Compared with anterior surgery, the posterior operation can be performed safely even though there is severe pleural adhesion.

The key to get good outcomes after this procedure is to minimize the risk of spinal reconstruction failure and TB recurrence by selecting appropriate cases that meet the following criteria: (i) monosegment spinal TB without a big gravitation abscess; and (ii) the TB lesion can be removed completely. Additionally, the patient should meet at least one of the following conditions: (i) bone destruction with collapse of the vertebral body and spinal instability; (ii) compression of the spinal cord or spinal nerve root by an abscess; and (iii) obvious or progressive spinal deformity.

## Conclusions

For monosegment spinal TB without a big gravitation abscess, compared with anterior surgery it safer, less traumatic and better deformity correction can be achieved by performing internal fixation, debridement and interbody bone graft *via* a posterior approach only.

## Supporting information

Supporting Information
